# Zinc-Loaded Black Phosphorus Multifunctional Nanodelivery System Combined With Photothermal Therapy Have the Potential to Treat Prostate Cancer Patients Infected With COVID-19

**DOI:** 10.3389/fendo.2022.872411

**Published:** 2022-03-31

**Authors:** Linfeng Li, Baotong Zhou, Haoyang Xu, Hailin Shi, Li Gao, Bo Ge

**Affiliations:** ^1^ Department of Urology, Affiliated Hospital of Guilin Medical College, Guilin, China; ^2^ The Second Affiliated Hospital of Guilin Medical University, Guilin, China

**Keywords:** COVID-19, zinc, prostate cancer, black phosphorus, photothermal therapy, immungentic cell death

## Abstract

Since 2019, coronavirus disease 2019 (COVID-19) has swept the world and become a new virus threatening the health of all mankind. The survey found that prostate cancer accounts for one in three male cancer patients infected with COVID-19. This undoubtedly makes prostate cancer patients face a more difficult situation. Prostate cancer is the second most harmful malignant tumor in men because of its insidious onset, easy metastasis, and easy development into castration-resistant prostate cancer even after treatment. Due to its high immunogenicity and a small number of specific infiltrating T cells with tumor-associated antigens in the tissue, it is difficult to obtain a good therapeutic effect with immune checkpoint blocking therapy alone. Therefore, in the current study, we developed a platform carrying Doxorubicin (DOX)-loaded black phosphate nanometer combined with photothermal therapy (PTT) and found this drug combination stimulated the immungentic cell death (ICD) process in PC-3 cells and DC maturation. More importantly, zinc ions have a good immunomodulatory function against infectious diseases, and can improve the killing ability of the nanosystem against prostate cancer cells. The introduction of Aptamer (Apt) enhances the targeting of the entire nanomedicine. We hope that this excellent combination will lead to effective treatment strategies for prostate cancer patients infected with COVID-19.

## Introduction

COVID-19 is a serious threat to the health of people around the world, especially cancer patients. Studies have highlighted the increased susceptibility of prostate cancer patients to COVID-19, leading to higher rates of hospitalization and mortality (1). This may be due to an imbalance in the immune system of cancer patients themselves, making it easier for COVID-19 to enter. According to the statistics released by the American Cancer Society in 2021, prostate cancer is the most common cancer in men, while it is the second place. Most common cause of cancer-related deaths (2). Prostate cancer is often overlooked because its early symptoms are insidious. When diagnosed, most of the patients have passed the early stage where surgery would have been an option and can only be treated using the androgen deprivation therapy (ADT). However, most of the patients develop castration-resistant prostate cancer or even neuroendocrine prostate cancer after 18-24 months of ADT, thereby suggesting a failure of the treatment (3). This extremely malignant progression may result from the lineage plasticity induced by adeno-PCa (prostate cancer) after androgen receptor (AR)-targeted therapy (4). Common treatment methods involving chemotherapy drugs have the disadvantages of toxic side-effects, the occurrence of multidrug resistance, and a high recurrence rate after surgery. It is, therefore, urgent to develop new effective strategies for the treatment of prostate cancer.

Recently, immunotherapy has shown remarkable efficacy in the treatment of malignant tumors (5). For instance, Chimeric antigen receptor T cell (CAR-T) therapy, which is mature passive immunotherapy, transforms T cells from ordinary “soldiers” into “super soldiers”, but these “super soldiers” do not seem to work well for solid tumors, including prostate cancer (6). They lead to a risk of an immune storm (7). In contrast, Sipuleucel-T (Provenge), the world’s only FDA-approved therapeutic vaccine for prostate cancer, is a classic example of active immunity (8). It has the disadvantages of weak anti-tumor reaction and poor universality, despite better overall survival (OS) (6). However, there is still hope for the development of a therapeutic vaccine for prostate cancer because prostate cancer has multiple tumor-associated antigens and is an indolent tumor, which provides sufficient time to stimulate an immune response (9).

Although prostate cancer has high immunogenicity, the tissue contains fewer infiltrating T cells specific to prostate cancer-associated antigens (10). Therefore, improving the presentation of prostate cancer-associated antigens by dendritic cells (DCs) and stimulating the body to produce more CD8+ T cells may be the key to successful immunotherapy. Studies have shown that chemotherapeutic drugs such as doxorubicin, photothermal therapy, and certain nanomaterials can induce immunogenic death (ICD) in tumor cells (11–13). As a special case of apoptosis, the body can produce a specific immune response to the antigens associated with dead cells (11) and stimulate damage-related molecular patterns (DAMPs), such as calreticulin (CRT) membrane transposition, which is the “eat me” signal, reminding DCs to recognize and phagocytize them (14, 15). Subsequently, ATP and high mobility group box 1 protein (HMGB1) are released (16), which promote the maturation of dendritic cells and the infiltration of CD8^+^ T cells into the tumor tissue (10).

DOX is the most widely used immunogenic death stimulant (14). However, the application of chemotherapy drugs alone has certain limitations, such as low induction efficiency and drug utilization rate (17). Therefore, we are trying to find a multifunctional drug carrier, which can not only enhance the ICD effect of chemotherapy drugs but also improve the target specificity.

A large number of studies have shown that nanocarriers have the advantages of large drug load, stable chemical properties, biological safety, and longer half-life in the blood (18, 19). Therefore, we speculate that the combination of nanocarriers and chemotherapeutic drugs may be beneficial. Black phosphorus two-dimensional nanosheets are excellent candidates due to their low cost, good biocompatibility, photoresponsiveness, and high drug loading rate (20). More notably, black phosphorus was found to perform well as an adjuvant in tumor photoimmunotherapy (21, 22). Photoimmunotherapy with black phosphorus as an adjuvant stimulates a stronger immune response *in vitro* and *in vivo* than monotherapy, inducing more cytokine release (23, 24).

Photothermal therapy (PTT) has received a lot of attention in recent years due to its noninvasiveness and high efficiency, but more importantly, due to its ability to induce the ICD process (25). Therefore, combining the photosensitizer black phosphorus with PTT can synergistically promote the ICD process.

Although black phosphorus is photostable, it is easily degraded when exposed to air (26). Because black phosphorus exposes a lone pair of electrons, it easily combines with oxygen and is removed by water, resulting in the structural destruction of black phosphorus (27, 28). The solution to this problem is to use an element to stabilize the lone pair (29). Zinc, as an easily available, non-toxic and low-cost raw material, is expected to be used as an excellent therapeutic adjuvant (30). Therefore, we added zinc ions to the nanosystem hoping that its occupation will improve the stability of black phosphorus. In addition, a large number of studies have found that the lack of trace element zinc has a loss of immune function. Adequate amounts of zinc maintain normal prostate function because it inhibits the activity of aconitase, thus depleting the energy of tumor cells (31). In our previous studies, it was confirmed that an increase in zinc ion levels has a killing effect on prostate cancer cells (32). However, the underlying killing mechanism is not clear.

Aptamer (APT) is a short oligonucleotide sequence or short polypeptide obtained *via in vitro* screening, can bind to the corresponding ligand with high affinity and strong specificity and has no immunogenicity (33, 34). AS1411 is an aptamer containing guanine, which inhibits the growth of tumor cells and has a high affinity to nucleolins (35). Nucleolins are highly expressed on the plasma membrane of prostatic cells (36). Therefore, the introduction of aptamers into the whole nanodrug delivery system can not only improve the precise targeting of drugs but also the tumor cell killing effect.

In the current study, we used black phosphorus nanosheets loaded with DOX and combined them with photothermal therapy to enhance the probability of ICD induction in prostate cancer cells. The addition of zinc ions not only stabilized black phosphorus but also enhanced the killing effect of the whole system, and the adaptor enhanced the targeting power.

## Materials and Methods

### Materials

The bulk black phosphorus (BP) was purchased from Nanjing Two-dimensional Nanotechnology Co., LTD (Nanjing, China). 1-Methyl-2-pyrrolidinone (NMP) and tris-(2-carboxyethyl)-phosphine hydrochloride (TCEP) were purchased from Sigma-Aldrich (St. Louis, MO, USA). Zinc acetate was obtained from Aladdin (Los Angeles, CA, USA). Aptamer (APT) AS1411 (5’-/SH/GGTGGTGGTGGTTGTGGTGTGGTGGTGG-3’) was customized by Guangzhou Ruibo Biotechnology Co., LTD (Guangzhou, China). Doxorubicin (DOX) was purchased from APExBIO (Houston, USA). PC-3 cells were kindly provided by the Stem Cell Bank, Chinese Academy of Sciences (Shanghai, China). Cell Counting Kit-8 (CCK-8) was provided by Dojindo (Japan). Dulbecco’s minimum essential medium (DMEM)/F12, penicillin-streptomycin liquid, and fetal bovine serum (FBS) were purchased from GIBCO (Grand Island, NY, USA). Alexa Fluor^®^ 488 Anti-Calreticulin antibody was purchased from Abcam (Cambridge, England). Anti-HMGB1 was obtained from Cell Signaling Technology (Boston, USA). Human HMGB1 ELISA Kit was obtained from Arigo Biolaboratories (Taiwan, China). ATP content detection kit was obtained from Solarbio (Beijing, China). Dendritic cells (DCs) were obtained from AllCells (San Francisco, USA). FITC anti-human CD11c Antibody, Alexa Fluor^®^ 647 anti-human CD80 Antibody, PE anti-human CD86 Antibody, and PerCP/Cyanine5.5 anti-human HLA-DR Antibody were provided by Biolegend (California, USA).

### Preparation of Black Phosphorus Nanosheets (BP NSs)

Black phosphorus nanosheets were obtained using an optimized liquid stripping method and step-by-step centrifugal screening for a suitable size.

First, 8 mg black phosphorus crystal powder was added to 24 mL NMP. Then, the mixture was subjected to ultrasonic treatment (800 W, on/off cycle time was 4 s/6 s) using a probe for 12 h in an ice bath, and the resultant brown suspension was centrifuged at 4000 rpm for 15 min. The supernatant was collected and centrifuged at 12000 RPM for 12 min to collect the sediment and obtain BP NSs, which were stored at 4°C.

### Preparation of BP-P-Apt

About 1 mg Apt-SH was dissolved into 1 mL Tris buffer (tris: 10 mM, pH = 7.4). Then, 2 mg NH_2_-PEG-Mal and 50 μg TCEP was added and stirred in the dark for 5 h to obtain NH_2_-PEG-Apt. Then, 2 mg BP NSs were added to the solution. After ultrasonic treatment in an ice bath for 20 min and stirring for 8 h, centrifugation was performed at 10000 rpm for 15 min to obtain BP-P-APT, and it was washed twice with deionized water.

### Preparation of BP-P-Apt-Zn

About 7 mg zinc acetate powder was added to 5 ml BP-P-APT suspension. The solution was subjected to ultrasound in an ice bath for 2 min, stirred for 1 hour, and centrifuged at 10000 rpm for 15 min, and the precipitate was washed with deionized water.

### Preparation of BP-P-Apt-Zn/DOX

About 2 mg BP-P-Apt-Zn/NSs was mixed in 2 mL DOX solution (1.5 mg/mL). The solution was stirred in the dark for 8 h. The precipitate was obtained by centrifugation at 10000 rpm for 15 min. It was then washed with deionized water and freeze-dried.

### Photothermal Conversion and Photostability

Temperature change and stability of black phosphorus were analyzed under different concentrations and laser powers using an infrared thermal imager (Ti400+, Fluke, USA). The concentrations of BP NSs and BP-P-Apt-Zn/Dox used were 50, 100, and 200 µg/mL, and they were irradiated for 10 min with an 808-nm laser at 0.5, 1, and 2 W/cm^2^ (Beijing Blueprint Photoelectric Technology Co., Ltd) to observe the temperature change. The irradiation was repeated 5 times for 10 min each time.

### Uptake of Drugs by Cells

About 1 × 10^6^ cells were inoculated in 6-well plates and anti-slip slide cell crawl plates were placed at the bottom of the wells. Complete medium(89%DMEM/F12+10%FBS+1% penicillin-streptomycin liquid), DOX, BP-P-Zn/DOX, and BP-P-Apt-Zn/DOX (DOX: 3 µg/mL) were added to separate wells. About 4 h later, the cells were washed with PBS thrice, fixed with 4% formaldehyde for 25 min, washed with PBS thrice again, stained with DAPI for 10 min, and observed using a fluorescence inverted microscope (Inverted fluorescence imaging microscope, Olympus, IX73 + DP80+ fluoview, Tokyo, Japan).

### Cell Culture

Human prostate cancer PC-3 cells were cultured in a T25 culture bottle containing 10% FBS, 1% penicillin-streptomycin liquid, and 89% DMEM/F12. The culture was maintained at 5% CO_2_ and 37°C. The medium was changed every other day.

### Cytotoxicity Assay

To evaluate the toxicity of zinc ion in PC-3 cells, PC-3 cells in the logarithmic phase were inoculated in 96-well plates (5 × 10^4^ cells per well) and then incubated with a medium containing zinc ions in different concentrations (1, 2.5, 5, 10, 20, and 30 µg/mL), separately. After 24 h, the cell survival rate was calculated using the CCK-8 assay.

The same method was used to evaluate the toxicity of DOX in PC-3 cells. The concentrations of DOX used were 0.125, 0.25, 0.5, 1, 2.5, 5, and 10 µg/mL.

To evaluate the killing effect of Apt and Zinc ions on PC-3, PC-3 cells were incubated with BP-P, BP-P-Apt, and BP-P-Apt-Zn at concentrations of 1, 10, 25, 50, 75, and 100 µg/mL for 24 h separately, and the cell survival rate was detected using the method mentioned above. The toxicity of BP-P-Apt-Zn/DOX and BP-P-Apt-Zn/DOX + laser (DOX: 0.25µg/mL;Zn:2.5µg/mL) in PC-3 cells in the photothermal treatment group was analyzed using the same method. After 5 h of drug treatment, the cells were irradiated with a laser at 808 nm and 1 W/CM^2^ for 12 min and then incubated. It is worth noting that because the absorbance of black phosphorus at 450 nm interferes with the OD value of the cells, 5 groups of drug-only media were used for normalization.

### Western Blotting Assay

Western blotting was performed to detect the expression of HMGB1 in the supernatant of PC-3 cells after 24 h of drug treatment (DOX: 3 µg/mL, BP-P: 40 µg/mL, BP-P+ laser: 40 µg/mL, BP-P-Apt-Zn/DOX: 40 µg/mL, and BP-P-Apt-Zn/DOX + laser: 40 µg/mL). The procedure was as follows: β-actin was used as the control protein. The supernatant was collected after 24 h of drug treatment and centrifuged at 12000 rpm for 15 min to remove cell debris. The samples were then mixed with SDS-PAGE protein loading buffer (Beyotime, China) and boiled for 5 min. About 20 µL of the samples were subjected to SDS-PAGE gel electrophoresis and then transferred to nitrocellulose membranes. Nitrocellulose membranes were incubated with rabbit monoclonal antibody (1:1000, CST, Boston, USA) and gently shaken at 4°C overnight. After 3 washes, the membranes were incubated with anti-Rabbit IgG and HRP-Linked Antibodies (1:2000, CST, Boston, USA) for 1 h at room temperature. After three washes, the HMGB1 protein bands were detected using Bio-RAD (California, USA), and the protein concentration was analyzed using ImageJ.

### Human HMGB1 ELISA

The HMGB1 protein level in the supernatant was detected according to the instructions provided with the Human HMGB1 ELISA Kit (Arigo Biolaboratories, Taiwan, China). This assay employs the sandwich enzyme immunoassay technique for the detection of Human HMGB1 in cell culture supernatant samples. The specific steps were performed according to the protocol provided by the manufacturer.

### Immunofluorescence Staining Assay

PC-3 cells were inoculated in 6-well plates at a density of 1 × 10^5^ per well. After 24 h, the drugs (DOX: 3 µg/mL, BP-P:40 µg/mL, BP-P+laser:40µg/mL, BP-P-Apt-Zn/DOX: 40 µg/mL, BP-P-Apt-Zn/DOX + laser: 40 µg/mL) were added. After 24 h, the cells were rinsed thrice, fixed with 4% paraformaldehyde for 20 min, rinsed thrice again, sealed with 10% bovine serum for 30 min, and incubated with Alexa Fluor^®^ 488 anti-calreticulin antibody (1:1000, Abcam, Cambridge, England) overnight, in the dark, at 4°C. The cells were then washed thrice, their nuclei were stained with DAPI, and the cells were observed using a fluorescence inverted microscope (Inverted fluorescence imaging microscope, Olympus, IX73 + DP80 + fluoview, Tokyo, Japan). The whole process was performed on ice.

### Flow Cytometry

PC-3 cells (1 × 10^5^) were inoculated in 6-well plates. The next day, drugs were added and incubated for 24 h (DOX: 3 µg/mL, BP-P:40 µg/mL BP-P+laser:40µg/mL, BP-P-Apt-Zn/DOX: 40 µg/mL, BP-P-Apt-Zn/DOX +laser: 40 µg/mL). After washing the cells thrice, they were digested with trypsin and washed again thrice with PBS containing 10% FBS. The precipitate was collected and incubated with Alexa Fluor^®^ 488 anti-calreticulin antibody (1:100, Abcam, Cambridge, England), in the dark for 30 min at 4°C. The samples were then washed thrice with PBS containing 10% FBS, centrifuged at 400 g for 5 min, resuspended in 500 µl PBS containing 10% FBS, and placed on ice. Each sample was analyzed using a flow cytometer (BD FASC Aria III, Becton Dickinson, California, USA) to determine the concentration of CRT on the cell membrane.

### ATP Release Assay

According to the manufacturer’s instructions, the concentration of ATP in the cell culture medium after different treatments was determined using UV spectrophotometry. Briefly, ATP was extracted from the sample. Due to the characteristic absorption peak of NADPH at 340 nm and the content of NADPH being proportional to the content of ATP, NADPH content was used to calculate ATP content.

### DCs Were Co-Incubated With Drug-Treated Cells

Human DCs were provided by AllCells (San Francisco, USA). After resuscitating the DCs, they were co-incubated with PC-3 cells treated with drugs for 24 h. Briefly, DCs were inoculated in 6-well plates with a density of 2 × 10^5^ cells per well, and then, drug-treated PC-3 cells with a density of 2 × 10^5^ per well were added and incubated for 30 h.

### Detection of DC Maturation

About 30 h later, the cells were centrifuged at 1200 rpm for 5 min thrice, and resuspended in PBS containing 10% FBS. They were incubated with FITC anti-human CD11c Antibody, Alexa Fluor^®^ 647 anti-human CD80 Antibody, PE anti-human CD86 Antibody, and PerCP/Cyanine5.5 anti-human HLA-DR Antibody (Biolegend, California, USA) at 1:100 (by volume) on ice for 30 min in the dark. After centrifugation thrice, they were suspended in 500 µl PBS and the matured DCs were detected using a flow cytometer (BD FASC Aria III, Becton Dickinson, California, USA).

## Results and Discussion

### Preparation and Characterization of BP-P-Apt-Zn/DOX

**Scheme 1:**
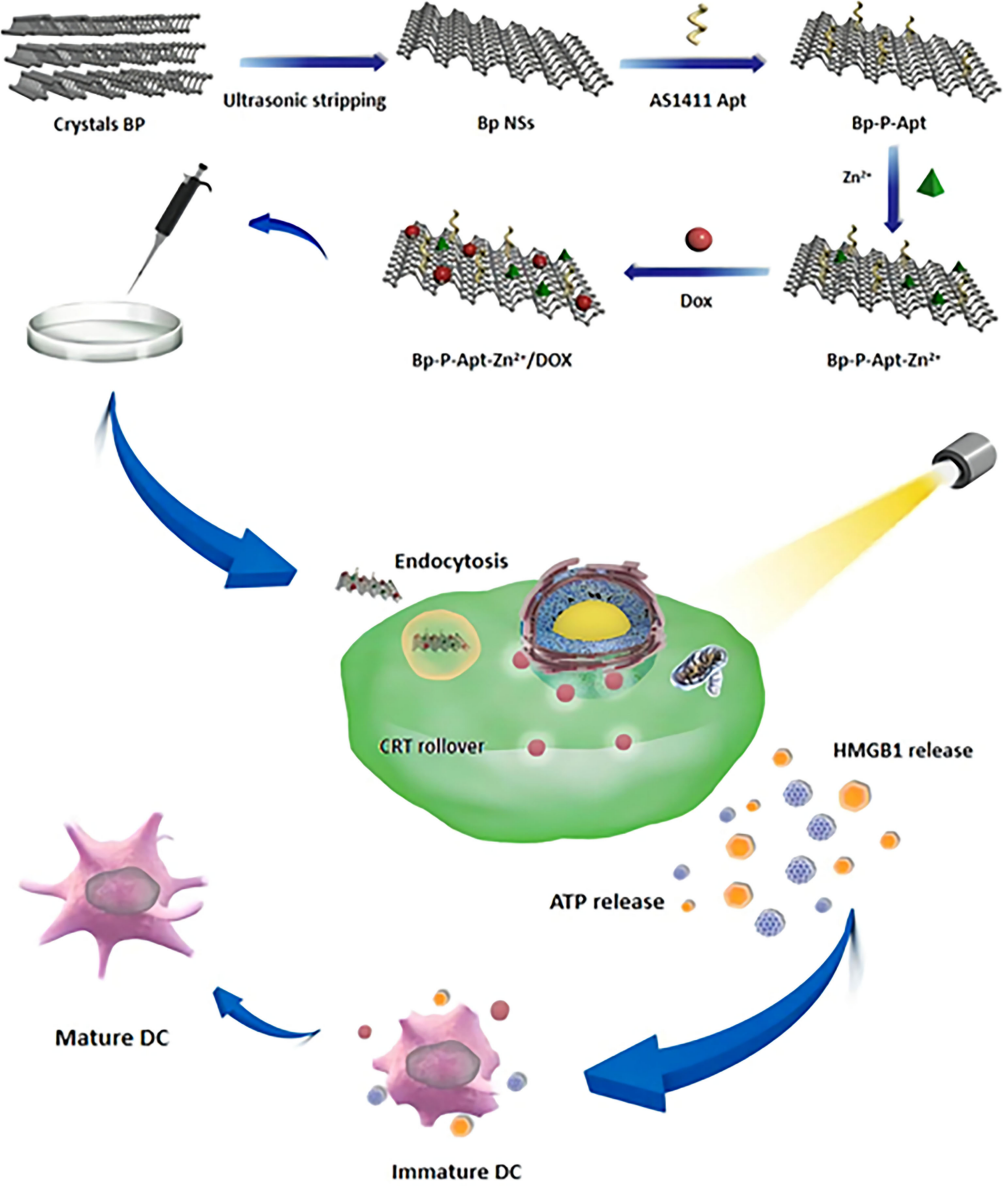
schematic diagram showing the preparation of BP-P-Apt-Zn/DOX and the ICD induction process.

The detailed synthesis process BP-P-Apt-Zn/DOX is shown in **Scheme 1**. First, the diameter of black phosphorus powder was changed to about 200 nm using an ultrasonic probe, Apt was loaded on BP nanosheets using electrostatic adsorption, then, the zinc ions were modified on BP-P-Apt using charge coupling, and finally, DOX was modified.

**Figure 1:**
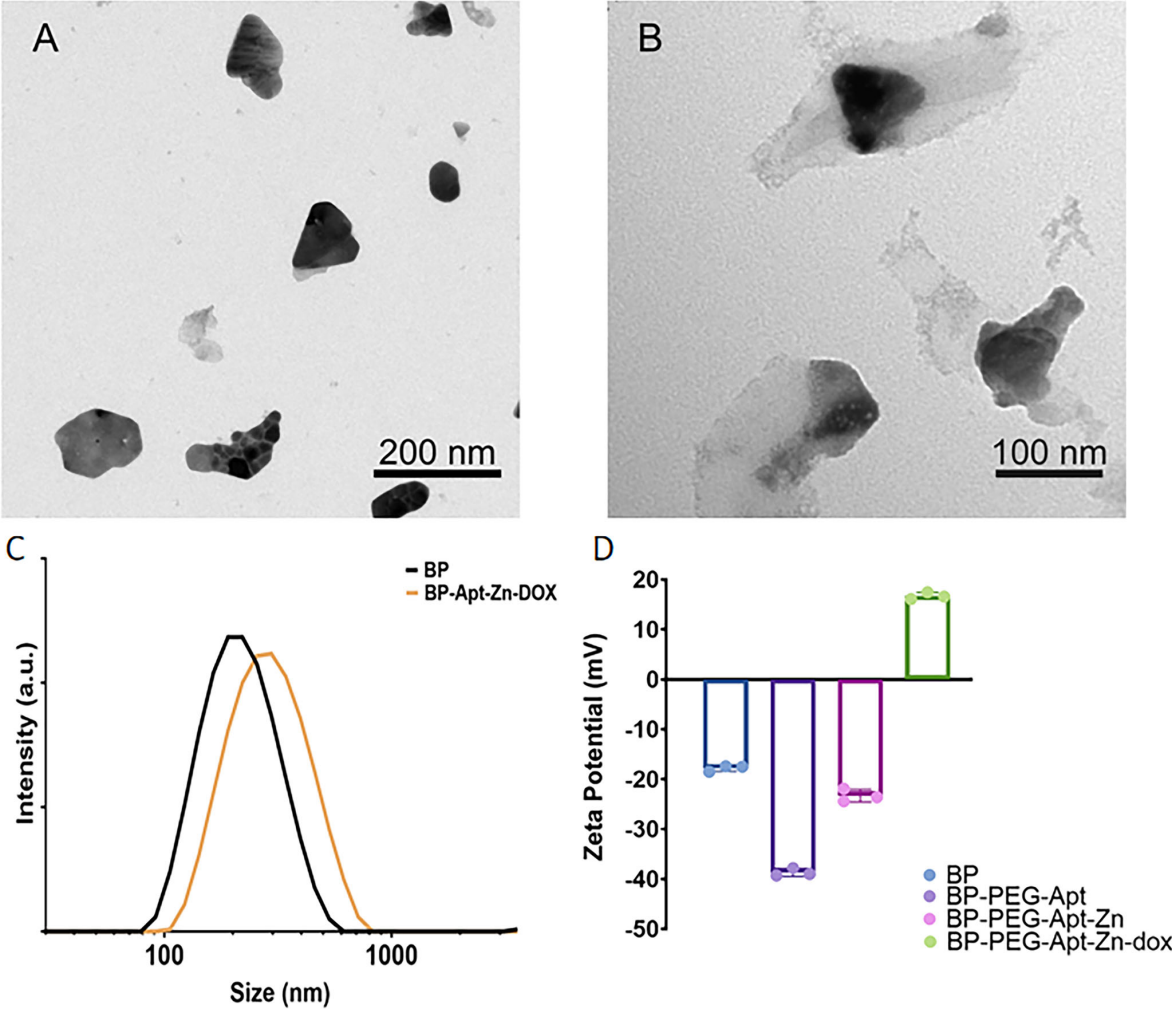
TEM images of **(A)** BP NSs, **(B)** BP-P-Apt-Zn/DOX. **(C)** Size distribution of BP and BP-Apt-Zn/DOX measured using DLS. **(D)** Zeta potentials of BP, BP-PEG-Apt, BP-PEG-Apt-Zn, and BP-PEG-Apt-Zn/DOX.

**Figures 1A, B** show the Transmission Electron Microscopy (TEM) photos before and after modification of BP NSs. As shown in **Figure 1A**, BP NSs were transparent and thin, indicating that the liquid stripping method had stripped off the black phosphorus and the stripping effect was good. BP NSs were about 100-200 nm in diameter. **Figure 1B** shows the appearance after modification using Zn, Apt, and DOX. It can be seen that the surface is covered with a transparent substance and the size is changed to 200-300 nm, indicating that the drug was successfully loaded. This was consistent with dynamic light scattering (DLS) results (**Figure 1C**).

As shown in **Figure 1D**, the initial Zeta potential of unmodified BP was about −17.8 mV, and after loading NH_2_-PEG-Apt, the Zeta potential changed to −38.7 mV (due to a large amount of PEG). With the coupling of Zn^2+^, the Zeta potential changed to −23.3 mV, and finally, when DOX was loaded, the Zeta potential changed to 16.7 mV.

**Figure 2:**
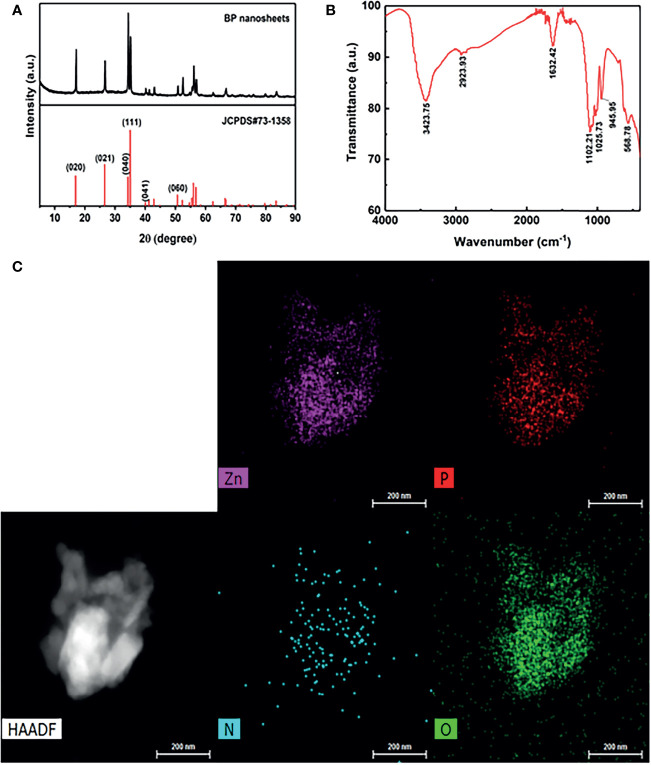
**(A)** X ray diffraction (XRD) of BP NSs. **(B)** Fourier Transform Infrared Spectroscopy (FTIR) of BP-P-Apt-Zn/DOX. **(C)** The high-angle annular dark-field (HAADF) SEM image of BP-P-Apt-Zn/DOX.

The crystal structure of BP NSs was characterized using X-ray diffraction. As shown in **Figure 2A**, compared to the standard PDF card JCPDS#73-1358, it was found that all the peaks of the prepared material were consistent with those of ortho-crystalline black phosphorus in the pure phase, and the characteristic peaks were not offset. The results showed that the lattice structure of BP was not damaged during ultrasonic stripping.

The surface group structure of BP-P-Apt-Zn/DOX was characterized using FTIR. As shown in **Figure 2B**, multiple peaks with high intensity appeared on the FTIR spectrum. At 3423.75 cm^-1^, multiple absorption peaks of hydrogen bond association formed by Apt, DOX, PEG, and NH_2_, including -OH stretching vibration absorption peak and -NH_2_ stretching vibration absorption peak. The stretching vibration absorption peak of C-H was at 2923.93 cm^-1^. The stretching vibration absorption peak of P = O was at 1632.42 cm^-1^. The stretching vibration absorption peaks of the PO4 group were at 1102.21 cm^-1^, 1025.73 cm^-1^, and 945.95 cm^-1^. The bending vibration absorption peak of the PO_4_ group was at 568.78 cm^-1^. PEG molecules chemically bind to BP by forming P-O-C bonds, which also led to the successful modification of BP surface with PEG.

To further identify the structure of BP-P-Apt-Zn/DOX, Scanning Electron Microscope (SEM) was used to analyze the nanostructure and characterize the element distribution in the nanostructure. The high-angle annular dark-field (HAADF) SEM image in **Figure 2C** shows the BP-P-Apt-Zn/DOX, and element mapping analysis was performed using the image. **Figure 2C** show SEM-mapping elements of P, Zn, N, and O, respectively. The SEM element mapping analysis revealed that the compositional distributions of the four elements (P, Zn, N, and O) in the BP-P-Apt-Zn/DOX are uniform, revealing the BP-P-Apt-Zn/DOX structure. DOX:C_27_H_29_NO_11_, PEG: HO(CH_2_CH_2_O)NH.

### Photothermal Conversion Properties

**Figure 3:**
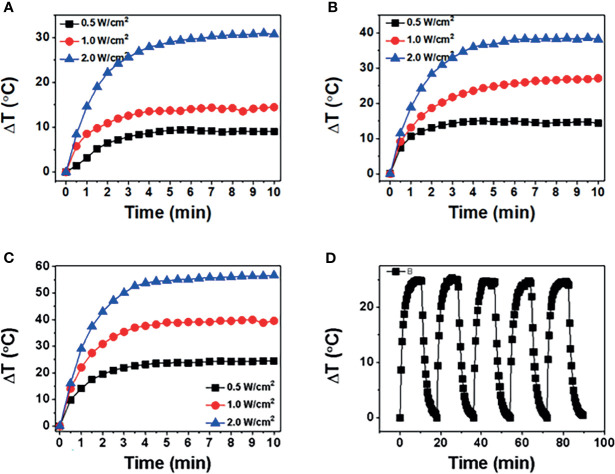
**(A**-**C)** show temperature of BP NSs at different powers (0.5, 1, and 2 W/cm^2^) and concentrations (50, 100, and 200 μg/mL). **(D)** BP NSs suspension (100 μg/mL) was irradiated using an 808-nm near-infrared laser with a power density of 1 W/cm^2^, and five laser switching cycles were performed.

The photothermal properties of BP NSs and BP-P-Apt-Zn/DOX were observed at different 808-nm laser powers and concentrations. The process was monitored using an infrared thermal imager. **Figures 3A–C** respectively show the temperature change curve of BP-NSs at the concentration of 50, 100 and 200μg/mL.After five cycles of irradiation with an 808-nm laser, the temperature changes in BP NSs (24.73°C ~ 25.09°C) (**Figure 3D**).

**Figure 4:**
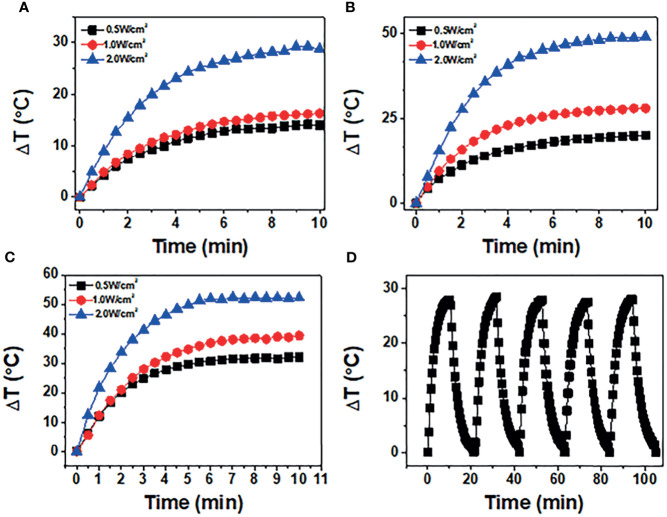
**(A**-**C)** show temperature of BP-P-Apt-Zn/DOX at different powers (0.5, 1, and 2 W/cm^2^) at concentrations (50, 100, and 200 μg/mL). **(D)** BP-P-Apt-Zn/DOX suspension (100 μg/mL) was irradiated using an 808-nm near-infrared laser with a power density of 1 W/cm^2^, and five laser switching cycles were performed.

**Figure 4A** shows the temperature changes at three powers when the concentration of BP-P-Apt-Zn /DOX is 50μg/mL. As shown in **Figure 4B**, when the concentration of BP-P-Apt-Zn/DOX is 100μg/mL and the power is 1W/cm2, the irradiation temperature can be increased by 28.03°C.When the concentration of BP-P-Apt-Zn/DOX was 200μg/mL, the temperature could be increased by 39.3°C (**Figure 4C**).It shows that the whole nanosystem has good photothermal conversion efficiency, and the increase of temperature is related to the concentration. Under the different powers, BP-P-Apt-Zn/ DOX at 100μg/mL could still reach 20.1°C at a power of 0.5 W/cm2 (**Figure 4B**).When the power is 2W/cm2, BP-P-Apt-Zn/DOX can reach 49.1°C (**Figure 4B**), and the increase of temperature is positively correlated with the power. As shown in **Figure 4D**, after five cycles of irradiation with an 808-nm laser, the temperature changes in BP-P-Apt-Zn/DOX (27.5°C ~ 28.5°C) were not significant, indicating good photostability.

### Cellular Uptake

**Figure 5:**
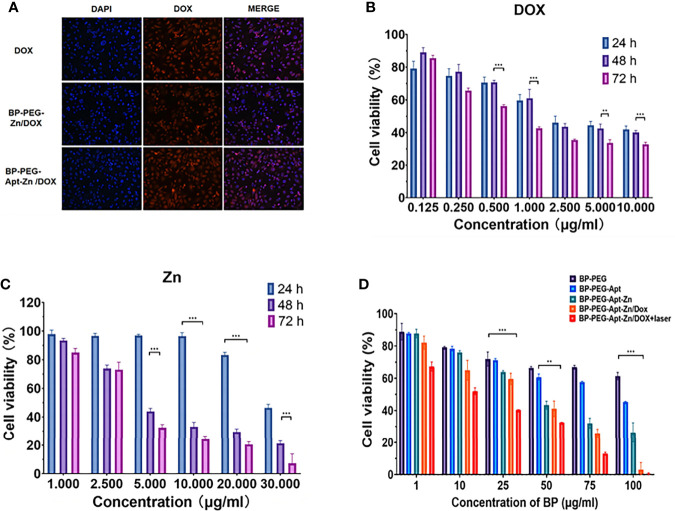
**(A)** Immunofluorescence of DOX, BP-P-Zn/DOX, and BP-P-Apt-Zn/DOX after 2 hours of treatment with PC-3 cells. **(B**, **C)** Cell survival rate of PC-3 cells treated with DOX, Zn at different concentrations after 24, 48, and 72 h. **(D)** Cell viability of PC-3 cells treated with different drugs. (**p < 0.01; ***p < 0.001).

To prove that the addition of Apt can improve the targeting efficiency of drugs, we used a fluorescence inverted microscope (Inverted fluorescence imaging microscope, Olympus, IX73 + DP80 + fluoview, Tokyo, Japan) to observe the uptake of DOX, BP-P-Zn/DOX, and BP-P-Apt-Zn/DOX by PC-3 cells. As shown in **Figure 5A**, compared with BP-P-Zn/DOX, the BP-P-Apt-Zn/DOX group showed stronger fluorescence, indicating that Apt-modified BP-P-Zn/DOX could be taken up in greater amounts by PC-3 cells. It is noteworthy that the fluorescence intensity of the only doxorubicin group was relatively high, probably due to the presence of the nuclear pore complex (37), which enables DOX to enter and exit cells efficiently. However, when only DOX is used, the long cycle capacity and biocompatibility of the nanodrug delivery platform is not achieved.

### Cytotoxicity Assay

We first explored the toxicity of Zn^2+^ and DOX alone in PC-3 cells. As shown in **Figure 5B**, the toxicity of DOX in PC-3 cells was time- and dose-dependent, with a survival rate of 33.7% at 72 h at a concentration of 5 μg/mL.

As shown in **Figure 5C**, the cytotoxicity of Zn^2+^ in PC-3 was also time- and dose-dependent. After 48 h of treatment, Zn^2+^ showed good cell destruction. When the concentration was only 5 µg/mL, the survival rate was 43.8% at 48 hours.

As shown in the **Figure 5D**, we analyzed the lethality of the addition of various components to the whole nanosystem towards PC-3 cells. It was observed that BP showed no obvious lethality towards PC-3 cells, the addition of Apt reduced the survival rate of cells, and BP-P-Apt-Zn had a strong killing effect.

However, the BP-P-Apt-Zn/DOX irradiation group had a stronger destruction effect than the non-irradiation group. When the concentration of BP-P-Apt-Zn/DOX was 50µg/mL, the survival rate of cells in the laser group was 32.6%, while that in the unlaser group was 41%.

### Induced Exposure of DAMPs

#### Release of HMGB1

**Figure 6:**
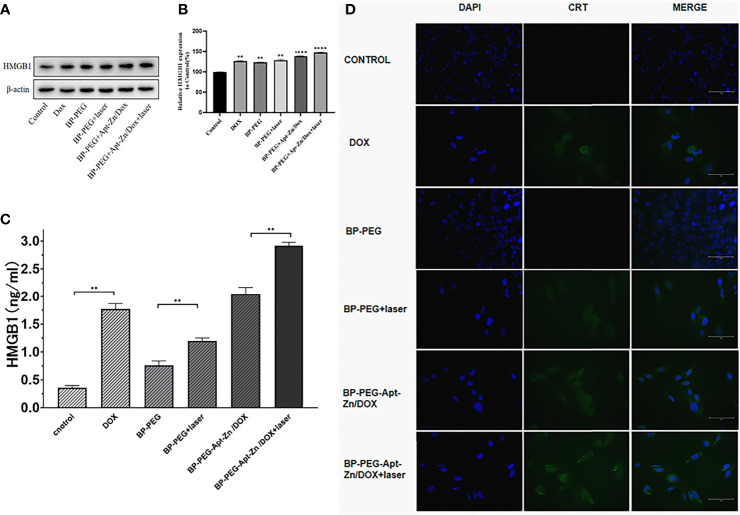
**(A, B)** The expression of HMGB1 in the supernatant of PC-3 cells after treatment with DOX, BP-P, BP-P+laser, BP-Apt-Zn/DOX, and BP-P-Apt-Zn/DOX+ laser (**p < 0.01; ****p < 0.0001). **(C)** The concentration of HMGB1 in the cell culture medium measured using ELISA (**p < 0.01). **(D)** Immunofluorescence assay of CRT in PC-3 cells after treatment.

Western blotting was used to analyze the level of HMGB1 released in the supernatant by the PC-3 cells after treatment with DOX, BP-P, BP-P+laser, BP-P-Apt-Zn/DOX, and BP-P-Apt-Zn/DOX+laser treatment for 24 h. As shown in **Figure 6A**, compared to the control group, the band strength in the DOX group increased and was 1.26 (**Figure 6B**) times that in the untreated group. The expression of HMGB1 in the laser-irradiated group was slightly higher than that in the non-irradiated group. Explain the effect of laser on the increase in the release of HMGB1. The band intensity of the BP-Apt-Zn/DOX + laser group was the strongest, which was 1.47 times that of the untreated PC-3 cells, indicating the synergistic effect of BP, DOX, and laser on the release of HMGB1 (**Figure 6B**). ser (*, p < 0.05; **, p < 0.01; ***, p < 0.001).

The HMGB1 ELISA kit was used to measure the released HMGB1 in the supernatant of the PC-3 cells treated with DOX, BP-P, BP-P+laser, BP-P-Apt-Zn/DOX, and BP-P-Apt-Zn/DOX + laser. As shown in **Figure 6C**, the concentration of released HMGB1 in the control group was 0.36 ng/mL, and that in the DOX group was 1.771 ng/mL, suggesting that DOX significantly increased the release of HMGB1. Similarly, the expression of HMGB1 in the laser-irradiated group increased compared to the non-irradiated group, indicating the positive effect of laser on HMGB1. The concentration of the released HMGB1 in the supernatant of the PC-3 cells after the treatment with BP-P-Apt-Zn/DOX + laser was the highest (2.916 ng/mL). Consistent with western blotting results, BP-P-Apt-Zn/DOX + laser increased the release of HMGB1.

### CRT Membrane Transposition

The transfer of CRT to the cell membrane was observed using the immunofluorescence assay. There was no expression of CRT on the membranes of the cells in the BP-P and control groups (**Figure 6D**), indicating that BP alone had no effect on the expression of CRT on the membrane. Weak green fluorescence of CRT was observed on the membranes in the DOX group, and an increased green fluorescence of BP-PEG was observed in the laser-irradiated group compared to the non-irradiated group. The fluorescence intensity in the BP-P-Apt-Zn/DOX + laser group was slightly higher than that in the BP-P-Apt-Zn/DOX group, but the difference was not significant. The combined application of BP, laser, and DOX played the most significant role in the transfer of CRT to the cell membrane.

**Figure 7:**
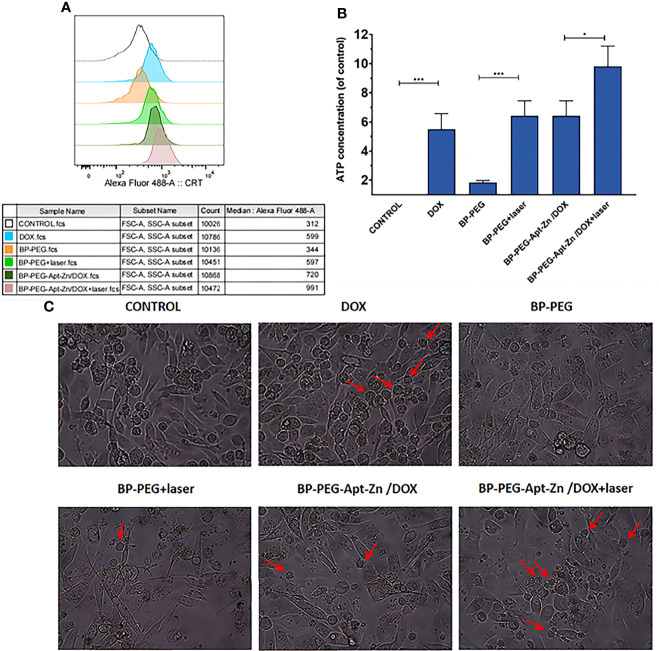
**(A)** Average fluorescence intensity of CRT on PC-3 cell surface after drug. **(B)** ATP expression in the cell culture medium after drug treatment. (*p < 0.05; ***p < 0.001). **(C)** PC-3 cells after treatment with drug and immature DCs for 24 h.(The red arrows indicate that immature DCs have developed “dendritic” structures, indicating that they have become mature DCs).

The average fluorescence intensity in CRT-positive cells was quantitatively analyzed using flow cytometry. As shown in **Figure 7A**, the CRT fluorescence intensity in the BP-Apt-Zn-Dox + laser group was about three times that in the control group. The laser-irradiated group also showed an excellent CRT fluorescence intensity (**Figure 7A**), which was consistent with the analysis of immunofluorescence. As can be seen in **Figure 7A**, the DOX group showed stronger fluorescence of CRT compared with the control group.

### Release of ATP

The release of ATP was measured using UV spectrophotometry. As shown in **Figure 7B**, the release of ATP in the extracellular fluid of PC-3 cells treated with DOX increased 5.5 times compared to that in the untreated group, while the release of ATP in the DOX group combined with the whole nanomedicine system increased 9.8 times, which was approximately consistent with the results of CRT and HMGB1. Similarly, the laser-irradiated group also showed significant enhancement.

### Maturation of DC Cells

To explore whether the nanoparticle drug platform-induced apoptosis of PC-3 cells can promote the maturation of anthropogenic DC cells to further induce an immune response, drug-treated PC-3 cells were co-incubated with anthropogenic DCs for 24 h. The maturation of DCs before and after drug treatment and in different drug groups was observed using an inverted microscope. As shown in **Figure 7C**, immature human DCs in their original state are round and have no dendrite structure. However, several DC suspension cells with the dendritic structure were observed in the DOX, laser, and all nanodrug groups, suggesting that the laser effect and the whole nanodrug platform could stimulate DC maturation.

**Figure 8:**
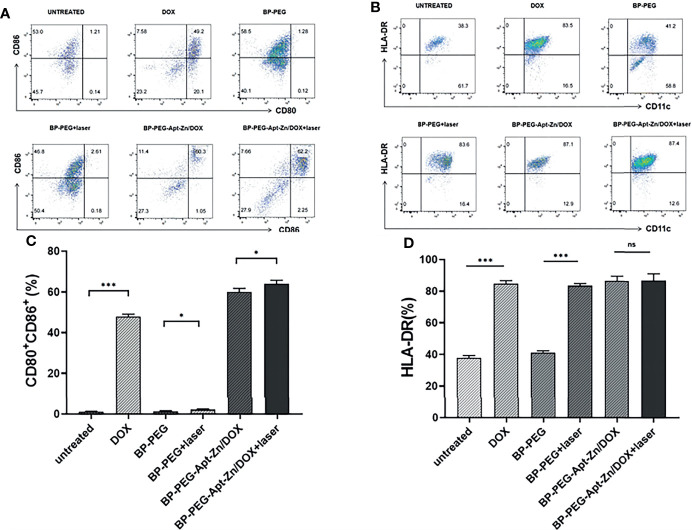
**(A)** The expression of CD80 and CD86 co-expressed DCs. **(B)** HLA-DR expression. **(C)**The expression of CD80 and CD86 co-expressed DCs. **(D)** HLA-DR expression. (*p < 0.05; ***p < 0.001; ns, no significance).

To further verify the maturation of DCs, flow cytometry was used to analyze the expression of related markers in dendritic cells after co-incubation. We first treated the cells with FITC anti-human CD11c to label the DCs, and then analyzed their CD80 and CD86 co-expression and HLA-DR expression. As shown in **Figure 8A**, the expression of CD80 and CD86 in the DOX group was significantly higher than that in the untreated group. There were no significant changes observed in the BP-P and BP-P+laser groups, while the expression of CD80 and CD86 in BP-P-Apt-Zn/DOX +laser and non-laser groups significantly increased by 62.2% and 60.3%, respectively. The expression of HLA-DR is shown in **Figure 8B**. Its expression in DOX and laser groups increased significantly, while there was no significant difference between the BP-P and untreated groups. **Figures 8C, D** shows the results of quantitative analysis of CD80 and CD86 are co-expressed and HLA-DR expression in dendritic cells. Flow cytometry analysis results were consistent with the microscopy results.

## Discussion

The COVID-19 pneumonia caused by SARS-COV-2 is severe. By February, 390 million cases were confirmed globally, with 2.44 million new cases reported daily. The economic losses and social burden caused by the epidemic are an unbearable disaster for every country, especially non-developed countries. The highly infectious and stealthy nature of the omicron variant makes COVID-19 more difficult to control (38). Multiple clinical studies have found significantly higher rates of hospitalization, morbidity and mortality in patients with solid tumors (prostate cancer) infected with COVID-19 (39). This may be due to abnormal immune status in solid tumor patients, coupled with radiotherapy and chemotherapy, the immunity is further reduced (40). Making it easier for COVID-19 to sneak in.

A large number of studies have confirmed the importance of trace element zinc for the development and maintenance of normal functions of immune system (41). Zinc deficiency can lead to a decrease in the number and function of immune cells, as well as an increased risk of tumors and infections (42). For prostate cancer patients with zinc deficiency, zinc ion supplementation can inhibit the activity of aconitase and inhibit the proliferation and invasion of tumor cells (43).

In the past decade, advances in tumor immunotherapy have changed the therapeutic landscape for solid malignancies. Prostate cancer, as a “cold” tumor, is challenging to diagnose and treat because of its indolence. Several Phase I and II trials evaluating programmed death receptor 1 (PD-1) inhibitors have shown a weak effect on metastatic castration-resistant prostate cancer (44). The only prostate cancer vaccine, Sipuleucel-T, was approved by the US FDA in 2010 for the treatment of asymptomatic or mild mCRPC, but it is not popular (45). Studies have shown that the ICD process of tumor cells and the release of DAMP can stimulate the anti-tumor immune response. CRT promotes the uptake of tumor cell membrane fragments by DCs. HMGB1 can bind toll-like receptor 4 (TLR4) to stimulate the immune response. ATP acts as a homing signal to activate the NLRP3 inflammasome (46).

Although DOX is not the most sensitive chemotherapy agent for prostate cancer, it is the most widely used immunogenic death stimulant (17). It certainly promotes ICD, a well-defined type of apoptotic process (47). Released DAMPs can be recognized by pattern recognition receptors (PRRs) and induce antigen-presenting cells(APC) to activate, differentiate, and mature (48). In addition, it promotes the release of interferon I, promotes the recruitment of antigen presenting cells and T cells, and further activates specific immune effects (49).

At present, there have been many milestone work on nanomedicine carriers. It is estimated that half of all drugs will be loaded with nanocarriers by 2050 (50, 51). Patisiran (ONPATTRO™), a double-stranded small interfering RNA encapsulated in lipid nanoparticles, is the first novel RNAi (interfering/silencing RNA) drug approved by the FDA for the treatment of amyloid multiple neuropathy (FAP) (52). However, it is prone to automatic oxidation, which leads to reduced fluidity of cell membrane and toxicity after aggregation and precipitation (53). As a kind of inorganic nanoparticles, Au NPs are more widely used in detection technology and biosensing. Due to the high cost of preparation, the application of Au Nps drug carrier cannot achieve universal applicability (54). The low cost, good biocompatibility, excellent photothermal conversion performance and good stability of modified black phosphorus made us choose it as our immune adjuvant. In the previous research, we developed DOX loaded black phosphorus nanomaterial and applied for relevant patents. Therefore, black phosphorus is still our first candidate material in this study. On the basis of previous research, the preparation process was optimized and its properties were more accurately characterized.

Our study introduced zinc ions into the nanodrug system, which not only improves immune function but also improves the ability to kill tumor cells. Moreover, BP-P-Apt-Zn/DOX combined with PTT promote ICD process and DCs maturation. Our findings are conducive to further activation of initial T cells, thus initiating, regulating, and maintaining the central link of the immune response.

Despite this initial success, there are a few shortcomings of our study because. Firstly, we used human PC-3 cells and human DCs and did not confirm our results in a prostate cancer model *in vivo*. In addition, we need to further study which specific pathway BP-P-Apt-Zn/DOX combined with PTT affects to induce the ICD process. Whether the ICD process can be further enhanced by attaching the nanodrug system to the anti-PD-1/PD-L1 antibody also needs to be further studied. Based on this study, we will use organoid or patient-derived xenotransplantation model (PDX) in the following plan to evaluate the exact efficacy of drugs *in vivo* and the ICD process.

## Conclusion

In summary, we successfully prepared the Zn-loaded black phosphorus nanodrug platform. The results proved that the introduction of Zn^2+^ improved the killing effect of the nanodrug system, the addition of Apt enhanced the drug targeting, and the combination of photothermal therapy further increased the killing effect of the whole nanodrug system. In addition, it was found that PTT combined with nanocarriers and loaded DOX not only enhanced toxicity but also promoted the ICD process and increased the maturation of DCs, thereby inducing an immune response. We hope this excellent combination will introduce prostate cancer vaccines to enhance their ability to activate the immune system. More importantly, it is hoped that the introduction of zinc will provide a new therapeutic strategy for prostate cancer patients infected with COVID-19.

## Data Availability Statement

The original contributions presented in the study are included in the article/supplementary material. Further inquiries can be directed to the corresponding authors.

## Author Contributions

LL as the first author of this paper was mainly responsible for the overall implementation of the experiment, data analysis, article writing and analysis of the experimental results. LG as the corresponding author was mainly responsible for the study design and experimental master plan as well as the review of the final paper. BG as the co-corresponding author, assisted LG to complete the overall planning of the whole experiment. BZ as the second author of this paper is mainly responsible for data processing. HX as the third author of this paper, mainly assisted LL in conducting experiments and analyzing data. HS as the fourth author was mainly responsible for assisting the experiment. All authors contributed to the article and approved the submitted version.

## Funding

This work was financially supported by the Guangxi Natural Science Fund (2018GXNSFAA281270, 2019GXNSFAA185034, 2020GXNSFAA238002), the National Natural Science Foundation of China (82060146), and the Young and middle-aged faculty research ability Enhancement project of Guilin Medical College (2018glmcy033).

## Conflict of Interest

The authors declare that the research was conducted in the absence of any commercial or financial relationships that could be construed as a potential conflict of interest.

## Publisher’s Note

All claims expressed in this article are solely those of the authors and do not necessarily represent those of their affiliated organizations, or those of the publisher, the editors and the reviewers. Any product that may be evaluated in this article, or claim that may be made by its manufacturer, is not guaranteed or endorsed by the publisher.
